# Determinants of hyperuricemia in non-dialysed chronic kidney disease patients in three hospitals in Cameroon

**DOI:** 10.1186/s12882-018-0959-5

**Published:** 2018-07-09

**Authors:** Marie Doualla, Marie Patrice Halle, Jude Moutchia, Steve Tegang, Gloria Ashuntantang

**Affiliations:** 1 Faculty of Medicine and Biomedical Sciences, University of Yaoundé I, Yaoundé, Cameroon; 2 Douala General Hospital, P.O. Box 4856, Douala, Cameroon; 3 Faculty of Medicine and Pharmaceutical Sciences, University of Douala, Douala, Cameroon

**Keywords:** Chronic kidney disease, Hyperuricemia, Glomerular filtration rate, Proteinuria, Cameroon

## Abstract

**Background:**

Chronic kidney disease (CKD) poses a substantial health burden in sub-Saharan Africa, with risk factors ranging from communicable to non-communicable diseases. Hyperuricemia has been recently identified as a factor of progression of CKD. Identifying factors associated with hyperuricemia in CKD patients would help determine interventions to reduce CKD mortality, particularly in resources limited countries. We sought to determine the prevalence and factors associated with hyperuricemia in non-dialysed CKD adult patients in Cameroon.

**Methods:**

This was a cross-sectional study of non-dialysed CKD patients, conducted in 3 referral nephrology units in Cameroon. Relevant clinical and laboratory data were collected using interviewer-administered questionnaires. Serum uric acid, spot urine protein and spot urine creatinine were assessed. Associations between variables were assessed using multivariate analysis. Level of statistical significance was set at α < 0.05.

**Results:**

A sample of 103 participants was included. Mean age of study participants was 55.78 ± 12.58 years, and 59.3% were men. Sixty-nine (67%) had hyperuricemia. Patient’s age (OR: 1.08, 95% CI: 1.03–1.13), estimated glomerular filtration rate (OR: 0.94, 95% CI: 0.90–0.98), spot urine protein-creatinine ratio (OR: 1.83, 95% CI: 1.07–3.12), no hypertension (OR: 0.09, 95% CI: 0.02–0.46), urate lowering therapy (OR: 4.99, 95% CI: 1.54–16.16), loop diuretics (OR: 3.39, 95% CI: 1.01–11.42), obesity (OR: 6.12, 95% CI: 1.15–32.55) and no anaemia (OR: 0.04, 95% CI: 0.00–0.29) were independently significantly associated with hyperuricemia.

**Conclusions:**

In this sample of non-dialysed CKD patients in Cameroon, about 7 out of 10 had hyperuricemia. Hyperuricemia was independently associated with patient’s age, estimated glomerular filtration rate, spot urine protein-creatinine ratio, hypertension, urate lowering therapy, loop diuretics, obesity and anaemia. More studies are required to establish causal relationships between these associations.

## Background

Chronic kidney disease (CKD) poses a substantial health burden in sub-Saharan Africa (SSA), with an estimated prevalence of 13.9% [1]. Risk factors of CKD in SSA range from communicable to non-communicable diseases [1, 2]. It is estimated that, by 2030, more than 70% of patients with end-stage renal disease (ESRD) will reside in low-income countries, such as countries of SSA [1]. In 2010, out of the 2.6 million people projected worldwide who accessed renal replacement therapy (RRT), only about 7% were from low-income and lower-middle income countries [3, 4]. In Cameroon, the morbidity and mortality of CKD is high, especially amongst patients on haemodialysis [5–7]. Emphasis should therefore be on prevention, early detection and managing factors of progression of CKD, in order to curb incidence of ESRD, while improving access to RRT in SSA [3, 8, 9].

Hyperuricemia has been recently identified as a factor of progression of CKD [10–12]. Findings from some observational studies show that increased serum uric acid is associated with progression of CKD in various subjects [13–18], and is highly associated with ESRD [19–22]. Also, findings from interventional studies show that lowering serum uric acid slows down CKD progression [23–25]. Although hyperuricemia is a recognised feature of CKD, studies evaluating its prevalence in CKD are relatively sparse in SSA. Reported prevalence of hyperuricemia in CKD in SSA range from 15.2 to 47.5% [26, 27].

Hyperuricemia in CKD has been mostly attributed to declining renal function [28]. However, in clinical practice, raised serum uric acid level in CKD is associated with a combination of several other factors, as not every CKD patient presents with hyperuricemia. In non-CKD subjects, factors associated with hyperuricemia include obesity, dyslipidaemia, alcohol consumption, fructose-rich and purine-rich diets, genetics and physical inactivity [29–31]. In CKD patients, factors associated with hyperuricemia have not been fully elucidated.

We therefore sought to determine the prevalence and factors associated with hyperuricemia in non-dialysed adult CKD patients in Cameroon, in order to generate findings that would help determine interventions to reduce CKD mortality, particularly in resources limited countries.

## Methods

### Study population and sampling

This was a cross sectional study carried out in the nephrology units of 3 hospitals in Cameroon (Douala General Hospital [DGH], Yaoundé Central Hospital [YCH] and Yaoundé University Teaching Hospital [YUTH]) over a period of 6 months (01 February 2014 to 31 July 2014). DGH and YCH are level III (referral) hospitals while YUTH is a level II (secondary) hospital in the Cameroon healthcare system. These sites are referral nephrology centres and receive patients with kidney diseases from all regions of the country.

After prior ethical clearance from the Cameroon National Ethics Committee, we systematically sampled every non-dialysed CKD patients aged 20 years and above, followed up in the nephrology units of the 3 hospitals. We excluded patients with chronic liver diseases, haemoglobinopathies and/or cancers as reported in their medical charts, and patients who did not give consent.

### Data collection

Socio-demographic data (age, gender) and relevant clinical information (aetiology of CKD, comorbidities, dietary regimens, use of medications known to influence serum uric acid and creatinine levels) were recorded from the patient’s medical chart or obtained by interviewer-administered questionnaire. Anthropometric data including height and weight were obtained using a stadiometer (measured to the nearest 0.1 cm, without any foot or head wear) and a manual weighing scale (measured to the nearest 0.1 kg, with light clothing and no foot wear) respectively. Blood pressure was measured according to World Health Organisation (WHO) guidelines [32] using an automatic blood pressure machine (OMRON® M2, HEM-7121-E). The most recent (less than 3 months) values of haemoglobin, serum high-density lipoprotein cholesterol (HDL-c), serum low-density lipoprotein cholesterol (LDL-c), serum total cholesterol, serum triglycerides and plasma creatinine in patient’s medical charts were recorded.

Uric acid in the serum specimen was measured using the direct kinetic uricase method [33]. A clean-catch mid-stream urine specimen from all participants was collected for spot protein and creatinine assessment using the Benzethonium Chloride [34] and Jaffé method [35] respectively.

### Definitions and calculations

Estimated glomerular filtration rate (eGFR) was computed from serum creatinine using the Chronic Kidney Disease Epidemiology Collaboration (CKD-EPI) equation [36]. CKD was defined by eGFR < 60 ml/min per 1.73 m^2^ for more than 3 months [37]. CKD was classified according to Kidney Disease: Improving Global Outcomes (KDIGO guidelines) into Stage 1/G1 (eGFR ≥90 ml/min per 1.73 m^2^), Stage 2/G2 (eGFR = 60–89 ml/min per 1.73 m^2^), Stage 3/G3a (eGFR = 45–59 ml/min per 1.73 m^2^), Stage 3b/G3b (eGFR = 30–44 ml/min per 1.73 m^2^), Stage 4/G4 (eGFR = 15–29 ml/min per 1.73 m^2^), Stage 5/G5 (eGFR< 15 ml/min per 1.73 m^2^) [37]. Proteinuria was classified into normal or mild increase/A1 (spot urine protein-creatinine ratio [PCR] < 150 mg/g), moderate increase/A2 (spot urine PCR = 150–500 mg/g) and severe increase/A3 (spot urine PCR > 500 mg/g) [37].

Renal disease was Glomerular if manifesting with glomerular range proteinuria, and/or haematuria (deformed or red blood cells cast), associated with hypertension and/or oedema; Tubulo-interstitial if manifesting with sterile leucocyturia, and low urine specific gravity, associated with or without non-glomerular haematuria and proteinuria; Vascular if presence of hypertension, with moderate proteinuria and normal urine sediment. Mixed renal involvement was renal disease manifesting with a combination of 2 or more of glomerular, tubulo-interstitial and vascular involvement.

Hyperuricemia was defined as serum uric acid > 70 mg/l in males and > 60 mg/l in females [38]. Hypertension was defined as a systolic blood pressure ≥ 140 mmHg and/or a diastolic blood pressure ≥ 90 or current use of antihypertensive drugs. Diabetes was defined as history of diabetes confirmed in the patient’s medical records and/or current use of blood glucose control medications. Gout was defined as history of gouty arthritis confirmed in the patient’s medical records and/or current use of urate lowering therapy (ULT) against gout. Low protein, low-carb and low fat diet were defined as a dietary regimen poor in proteins (less than 30 g daily), sugars and lipids respectively, as prescribed by a dietician. Body mass index (BMI) was classified as; underweight (BMI < 18.5 kg/m^2^), normal weight (BMI = 18.5–24.9 kg/m^2^), overweight (BMI = 25–29.9 kg/m^2^) and obesity (BMI ≥ 30 kg/m^2^) [39]. Anaemia was defined as haemoglobin < 13.0 g/dl in males and < 12.0 g/dl in females [40]. Dyslipidaemia was defined as total cholesterol ≥240 mg/dl (6.21 mmol/l) or low-density lipoprotein cholesterol (LDL-c) > 160 mg/dl (4.14 mmol/l) or high-density lipoprotein cholesterol (HDL-c) < 40 mg/dl (1.03 mmol/l) or triglycerides ≥150 mg/dl (1.69 mmol/l) [41].

### Statistical analysis

Statistical analyses were done using Statistical Package for Social Sciences (SPSS), version 23 Inc., Chicago, Illinois, USA). Normality of continuous data was assessed using Shapiro-Wilk test. Continuous variables were summarised as means and standard deviations (SD), and medians and interquartile ranges (IQR) where appropriate. Categorical variables were summarised as frequencies and percentages.

One-Way ANOVA was used to compare means between more than 2 independent groups. Bivariate analyses were done using generalized linear models (using serum uric acid as outcome variable) and logistic regression (using hyperuricemia as outcome variable). After bivariate analyses, variables with *p* < 0.02 were included as adjustment variables in multivariate analyses. Continuous data were selected over categorical data for variables with both continuous and categorical data. In order to avoid multicollinearity, bivariate correlations were assessed between the predictor variables prior to multivariate analyses. Predictor variables which were highly significantly (*p* < 0.01) on bivariate correlation were excluded from the multivariate analysis. Furthermore, collinearity diagnostics were done to ensure that none of the predictor variables had Tolerance < 0.3 and/or Variance Inflation Factor > 3. There were missing data in lipid profile indices (Total Cholesterol [10 missing], HDL-c [11 missing], LDL-c [11 missing], and Triglycerides [14 missing]. Missing data where excluded from the analysis pairwise. Level of statistical significance was set at α < 0.05.

Sensitivity analyses were done, defining hyperuricemia as serum uric acid > 70 mg/l in males and > 60 mg/l in females or current use of ULT. The results were very similar to our current results.

## Results

Out of the 198 non-dialysed CKD patients aged 20 years and above who attended our study sites during the study period, 2 patients were excluded for chronic liver disease (liver cirrhosis), one patient excluded for haemoglobinopathy (sickle cell disease), and one patient excluded for cancer (prostate cancer). Of the 194 eligible CKD patients approached for participation in the study, 103 patients gave consent and were included into study; thus a participation rate of 53.1%.

### Baseline characteristics of study participants

Mean age of our study participants was 55.78 ± 12.58 years, and 61 (59.3%) were males. The median (IQR) eGFR was 17.00 (11.00) ml/min per 1.73m^2^, and median (IQR) spot urine PCR ratio was 803.0 (1120.0) mg/g. More than two-thirds of participants were at an advanced stage of CKD with 48% at stage 4 and 25.2% at stage 5. The most common comorbidities identified were hypertension (87.4%), diabetes (34.0%) and gout (21.4%). The types of renal involvement included; glomerular (60.2%), vascular (19.4%), tubulo-insterstitial (11.7%), and mixed (8.7%). Forty-two (40.2%) participants were on ULT (Allopurinol), 32 (31.1%) were on loop diuretics (Furosemide), while 5 (4.9%) were on angiotensin receptor blockers (Losartan). The mean serum uric acid of the study participants was 76.03 ± 20.50 mg/l. Table 1.

**Table 1:** Baseline characteristics of study participants

Variable	Total (*N* = 103)
Age (years), mean ± SD	55.78 ± 12.58
Age Strata, n (%)	
20–29 years	2 (1.9)
30–39 years	9 (8.7)
40–49 years	19 (18.4)
50–59 years	33 (32.0)
60–69 years	23 (22.3)
> 70 years	17 (16.5)
Male gender, n (%)	61 (59.2)
Duration of CKD diagnosis (months), mean ± SD	14.67 ± 18.05
CKD stage, n (%)	
Stage 1/G1	0 (0.0)
Stage 2/G2	4 (3.9)
Stage 3a/G3a	8 (7.8)
Stage 3b/G3b	15 (14.6)
Stage 4/G4	50 (48.5)
Stage 5/G5	26 (25.2)
Type of renal involvement, n (%)	
Glomerular	62 (60.2)
Tubulo-interstitial	12 (11.7)
Vascular	20 (19.4)
Mixed	9 (8.7)
Comorbidities, n (%)	
Hypertension	90 (87.4)
Diabetes	35 (34.0)
Gout	22 (21.4)
HIV	13 (12.6)
Dietary regimen, n (%)	
None	32 (31.1)
Low protein	46 (55.3)
Low-carb	52 (50.5)
Low fat	29 (28.2)
Medications, n (%)	
^a^Urate lowering therapy	42 (40.8)
^b^Loop diuretics	32 (31.1)
^c^Thiazide diuretics	1 (1.0)
^d^ARB	5 (4.9)
Systolic Blood Pressure (mmHg), mean ± SD	142.3 ± 30.3
Diastolic Blood Pressure (mmHg), mean ± SD	88.3 ± 21.3
Weight (kg), median (IQR)	75.00 (18.40)
Height (m), median (IQR)	1.66 (0.10)
BMI (kg/m^2^), median (IQR)	26.30 (6.04)
Haemoglobin (g/dl), mean ± SD	10.27 ± 1.76
Total Cholesterol (mmol/l), mean ± SD	2.06 ± 0.56
HDL-c (mmol/l), mean ± SD	0.55 ± 0.19
LDL-c (mmol/l), mean ± SD	1.25 ± 0.42
Tryglycerides (mmol/l), median (IQR)	1.04 (0.72)
Plasma creat (mg/dl), median (IQR)	3.65 (1.55)
eGFR (ml/min per 1.73m^2^), median (IQR)	17.00 (11.00)
Spot urine protein (mg/dl), median (IQR)	1.07 (1.06)
Spot urine creat (mg/dl), median (IQR)	1.47 (1.14)
Spot urine PCR (mg/g), median (IQR)	803.0 (1120.0)
Serum uric acid (mg/l), mean ± SD	76.19 ± 20.25
Hyperuricemia, n (%)	69 (67.0)

*CKD* Chronic kidney disease, *HIV* Human immuno-deficiency virus, *ARB* Angiotensin receptor blockers, *BMI* Body mass index, *HDL-c* high-density lipoprotein cholesterol, *LDL-c* low-density lipoprotein cholesterol, *Creat* Creatinine, *eGFR* Estimated glomerular filtration rate, *PCR* Protein-to-creatinine ratio, *SD* standard deviation, *IQR* interquartile range

^a^Allopurinol

^b^Furosemide

^c^Hydrochlorothiazide

^d^Losartan

### Prevalence of hyperuricemia in chronic kidney disease

The prevalence of hyperuricemia amongst our study participants was 67% (95% CI: 58.3–75.7%).

### Factors associated with hyperuricemia in chronic kidney disease

**Fig. 1:**
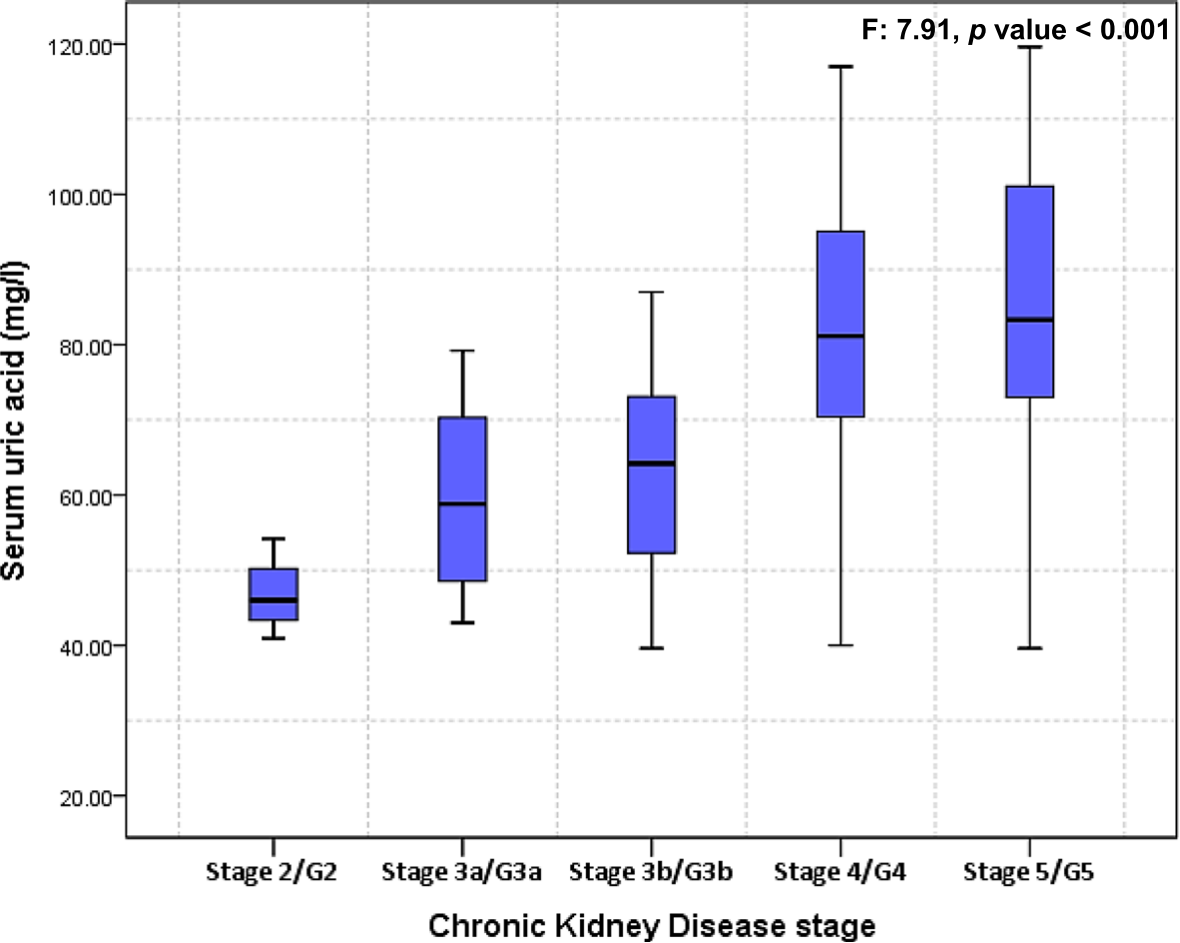
Boxplot of Serum uric acid versus Chronic Kidney Stage

**Fig. 2:**
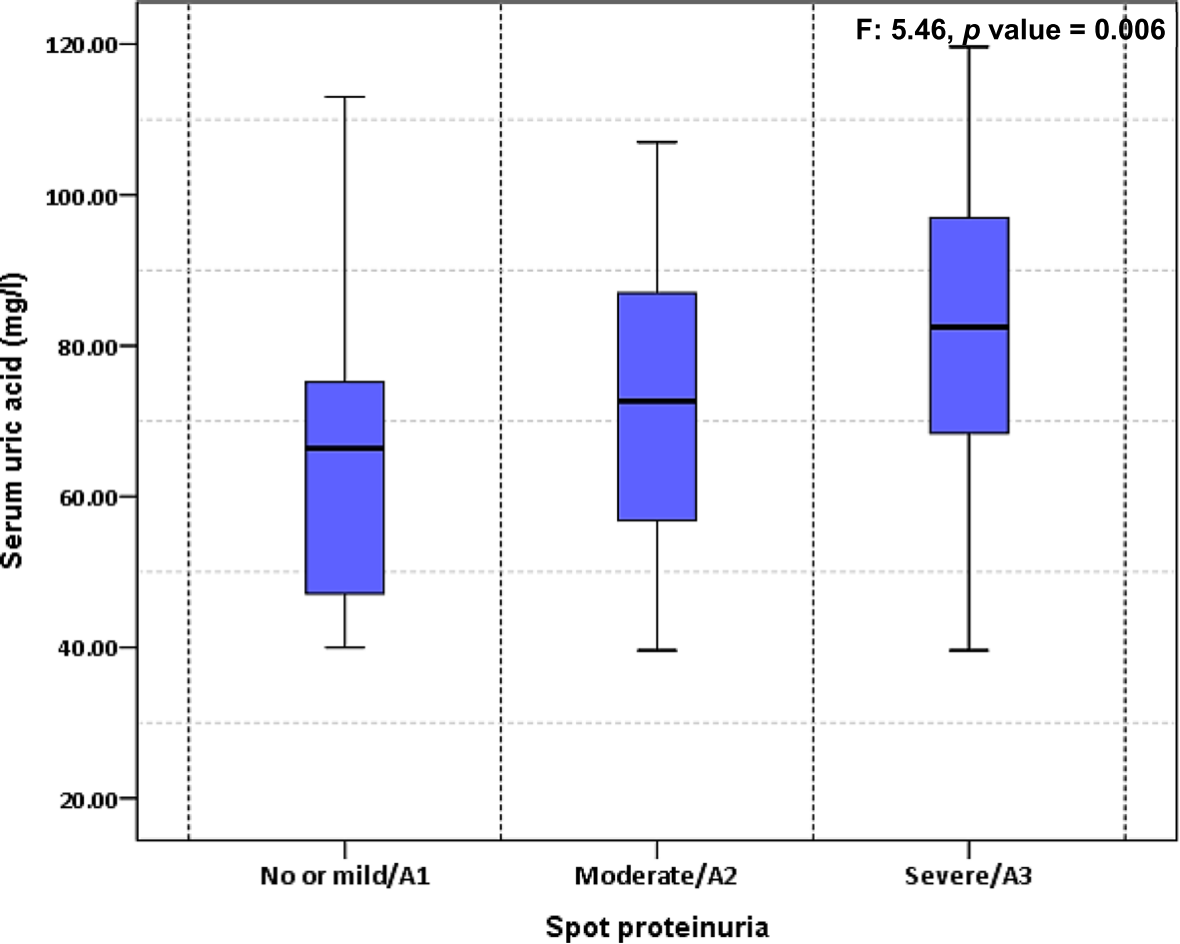
Box plot of Serum uric acid versus stage of proteinuria

On bivariate analysis, patient’s age (β: 0.54, 95% CI: 0.25–0.84), eGFR (β: -0.67, 95% CI: -0.91 - -0.43), CKD stage 4 (β: 20.55, 95% CI: 10.32–30.76) or CKD stage 5 (β: 20.82, 95% CI: 9.55–32.09), spot urine PCR (β: 3.76, 95% CI: 1.12–6.40), severe proteinuria (β: 16.51, 95% CI: 6.56–26.46), no hypertension (β: -17.83, 95% CI: -27.17 - -8.48), systolic (β: 0.25, 95% CI: 0.09–0.40) and diastolic (β: 0.58, 95% CI: 0.14–1.01) blood pressures, diabetes (β: 9.01, 95% CI: 0.87–17.14), low protein diet (β: -8.31, 95% CI: -16.07 - -0.55), low-Carb diet (β: 8.36, 95% CI: 0.64–16.07), ULT (β: 22.71, 95% CI: 16.00–29-42), loop diuretics (β: 11.94, 95% CI: 3.75–20.13), body mass index (β: 1.06, 95% CI: 0.07–2.05) and no anaemia (β: -27.42, 95% CI: (− 39.02 - -15.82) were significantly associated with serum uric acid. Mean serum uric acid significantly differed across CKD stages (F: 7.91, *p* value < 0.001) Fig. 1, and significantly differed by severity of proteinuria (F: 5.46, *p* value = 0.006) Fig. 2.

Also, on bivariate logistic regression, patient’s age (OR: 1.05, 95% CI: 1.02–1.09), eGFR (OR: 0.93, 95% CI: 0.89–0.96), CKD stage 4 (OR: 9.11, 95% CI: 2.01–33.21) or CKD stage 5 (OR: 6.67, 95% CI: 1.63–27.27), spot urine PCR (OR: 1.52, 95% CI: 1.01–2.30), severe proteinuria (OR: 4.71, 95% CI: 1.59–13.99), no hypertension (OR: 0.06, 95% CI: 0.01–0.30), systolic (OR: 1.03, 95% CI: 1.01–1.06) and diastolic (OR: 1.05, 95% CI: 1.00–1.11) blood pressures, low protein diet (OR: 0.42, 95% CI: 0.18–0.98), low fat diet (OR: 3.09, 95% CI: 1.06–9.02), ULT (OR: 6.71, 95% CI: 2.32–19.37), loop diuretics (OR: 3.73, 95% CI: 1.29–10.82), obesity (OR: 6.65, 95% CI: 1.36–32.61) and no anaemia (OR: 0.04, 95% CI: 0.00–0.29) were significantly associated with hyperuricemia.

On multivariate analysis, after adjusting for patient’s age, eGFR, spot urine PCR, systolic blood pressure, ULT, loop diuretics and haemoglobin level, patient’s age (β: 0.49, 95% CI: 0.24–0.74), eGFR (β: -0.36, 95% CI: -0.57 - -0.16), CKD stage 4 (β: 17.49, 95% CI: 9.52–25.45) or CKD stage 5 (β: 13.28, 95% CI: 4.41–22.16), spot urine PCR (β: 3.06, 95% CI: 1.09–5.04), severe proteinuria (β: 13.94, 95% CI: 4.92–22.95), no hypertension (β: -18.37, 95% CI: -28.67 - -8.06), systolic (β: 0.14, 95% CI: 0.02–0.25) and diastolic (β: 0.35, 95% CI: 0.03–0.66) blood pressures, ULT (β: 18.84, 95% CI: 12.98–24.71), loop diuretics (β: 6.62, 95% CI: 0.25–12.99) and no anaemia (β: -18.08, 95% CI: (− 27.55 - -8.61) were significantly associated with serum uric acid. Table 2.

**Table 2:** Factors associated with serum uric acid and hyperuricemia in Chronic Kidney Disease

Variable	Serum uric acid		Hyperuricemia	
	Bivariate analysis	^a^Multivariate analysis	Bivariate analysis	^a^Multivariate analysis
	β (95% CI)	β (95% CI)	cOR (95% CI)	aOR (95% CI)
Age	0.54 (0.25–0.84)	0.49 (0.24–0.74)	1.05 (1.02–1.09)	1.08 (1.03–1.13)
Male gender	0.15 (− 1.79–14.41)	1.96 (− 4.41–8.34)	0.85 (0.37–1.98)	0.58 (0.17–1.91)
Duration of CKD diagnosis (months)	− 0.01 (− 0.23–0.22)	−0.11 (− 0.26–0.05)	1.01 (0.99–1.04)	0.99 (0.96–1.03)
GFR (ml/min per 1.73m^2^)	−0.67 (− 0.91 - -0.43)	−0.36 (− 0.57 - -0.16)	0.93 (0.89–0.96)	0.94 (0.90–0.98)
CKD stage				
Stage 2/G2	− 15.08 (− 34.64–4.48)	2.68 (− 17.85–12.49)	–	–
Stage 3a/G3a	3.61 (− 11.61–18.83)	10.79 (− 1.16–22.75)	1.2 (0.20–7.18)	5.44 (0.45–65.66)
Stage 3b/G3b	Ref.	Ref.	Ref.	Ref.
Stage 4/G4	20.55 (10.32–30.76)	17.49 (9.52–25.45)	9.11 (2.01–33.21)	24.66 (3.63–167.63)
Stage 5/G5	20.82 (9.55–32.09)	13.28 (4.41–22.16)	6.67 (1.63–27.27)	7.73 (1.06–56.30)
Spot urine PCR (mg/g)	3.76 (1.12–6.40)	3.06 (1.09–5.04)	1.52 (1.01–2.30)	1.83 (1.07–3.12)
Proteinuria				
No or Mild/A1	Ref.	Ref.	Ref.	Ref.
Moderate/A2	8.81 (−3.08–20.70)	5.41 (− 5.26–16.07)	1.99 (0.57–6.90)	1.65 (0.39–6.90)
Severe/A3	16.51 (6.56–26.46)	13.94 (4.92–22.95)	4.71 (1.59–13.99)	6.27 (1.66–23.60)
Type of renal involvement				
Glomerular	Ref.	Ref.	Ref.	Ref.
Tubulointerstitial	−3.31 (− 15.63–9.02)	5.76 (− 3.47–14.99)	0.77 (0.22–2.72)	1.71 (0.30–9.67)
Vascular	7.36 (− 2.69–17.41)	4.39 (− 2.97–11.74)	1.10 (0.25–4.83)	2.25 (0.43–11.71)
Mixed	−8.92 (− 22.86–5.02)	−5.67 (− 15.70–4.36)	2.20 (0.65–7. 40)	1.57 (0.24–10.19)
No Hypertension	−17.83 (− 27.17 - -8.48)	−18.37 (− 28.67 - -8.06)	0.06 (0.01–0.30)	0.09 (0.02–0.46)
Systolic Blood Pressure (mmHg)	0.25 (0.09–0.40)	0.14 (0.02–0.25)	1.03 (1.01–1.06)	1.04 (1.01–1.07)
Diastolic Blood Pressure (mmHg)	0.58 (0.14–1.01)	0.35 (0.03–0.66)	1.05 (1.00–1.11)	1.06 (0.99–1.13)
Diabetes	9.01 (0.87–17.14)	4.34 (− 2.72–11.40)	2.09 (0.83–5.29)	1.40 (0.47–4.18)
HIV	−8.70 (− 20.45–3.04)	−5.15 (− 13.87–3.57)	0.76 (0.23–2.53)	1.37 (0.27–6.99)
Low protein diet	−8.31 (− 16.07 - -0.55)	−1.45 (− 7.70–4.80)	0.42 (0.18–0.98)	0.80 (0.29–2.21)
Low-Carb diet	8.36 (0.64–16.07)	−0.67 (− 7.18–5.84)	2.10 (0.91–4.86)	1.09 (0.41–2.91)
Low fat diet	6.20 (− 2.47–14.88)	−0.92 (− 7.16–5.32)	3.09 (1.06–9.02)	1.73 (0.44–6.81)
^b^Urate lowering therapy	22.71 (16.00–29-42)	18.84 (12.98–24.71)	6.71 (2.32–19.37)	4.99 (1.54–16.16)
^c^Loop diuretics	11.94 (3.75–20.13)	6.62 (0.25–12.99)	3.73 (1.29–10.82)	3.39 (1.01–11.42)
^d^Thiazide diuretics	4.82 (− 35.34–44.98)	−6.43 (− 34.72–21.86)	–	–
^e^ARB	−1.46 (− 19.78–16.87)	1.54 (11.69–14.77)	0.73 (0.12–4.57)	2.38 (0.08–69.14)
BMI (kg/m^2^)	1.06 (0.07–2.05)	0.49 (−0.35–1.32)	1.12 (0.99–1.26)	1.04 (0.89–1.21)
BMI class				
Normal	Ref.	Ref.	Ref.	Ref.
Overweight	4.99 (− 3.61–13.59)	5.52 (−1.38–12.41)	1.38 (0.57–3.35)	1.59 (0.56–4.53)
Obesity	11.29 (0.57–22.02)	4.34 (−4.48–13.16)	6.65 (1.36–32.61)	6.12 (1.15–32.55)
Haemoglobin (g/dl)	−2.82 (− 5.00 - -0.63)	−1.61 (− 3.26–0.04)	0.77 (0.61–0.99)	0.86 (0.64–1.15)
No anaemia	− 27.42 (− 39.02 - -15.82)	−18.08 (− 27.55 - -8.61)	0.04 (0.00–0.29)	0.04 (0.00–0.46)
Total Cholesterol (mmol/l)	4.85 (− 8.33–18.0)	9.24 (− 2.54–21.02)	1.64 (0.38–7.40)	1.05 (0.11–9.77)
HDL-c (mmol/l)	− 29.28 (− 67.02–8.46)	−4.16 (− 33.53–25.22)	0.11 (0.01–7.30)	0.01 (0.00–30.34)
LDL-c (mmol/l)	4.81 (−12.63–22.25)	−1.06 (− 14.88–12.76)	1.81 (0.24–13.82)	0.38 (0.21–6.91)
Triglycerides (mmol/l)	5.30 (−7.67–18.27)	3.76 (− 5.59–13.11)	1.34 (0.32–5.67)	1.40 (0.96–12.34)
Dyslipidaemia	10.62 (−4.60–25.85)	11.23 (− 1.58–24.03)	4.25 (0.82–22.13)	2.13 (0.13–34.26)

*cOR* Crude odds ratio, *aOR* adjusted odds ratio, *eGFR* Estimated glomerular filtration rate, *PCR* Protein-to-creatinine ratio, *CKD* Chronic kidney disease, *HIV* Human immuno-deficiency virus, *ARB* Angiotensin receptor blockers, *BMI* Body mass index. *HDL-c* high-density lipoprotein cholesterol, *LDL-c* low-density lipoprotein cholesterol

^a^Multivariate analysis adjusted for patient’s age, eGFR, spot urine PCR, systolic blood pressure, ULT, loop diuretics and haemoglobin level

^b^Allopurinol

^c^Furosemide

^d^Hydrochlorothiazide

^e^Losartan

On multivariate logistic regression, after adjusting for patient’s age, eGFR, spot urine PCR, systolic blood pressure, ULT, loop diuretics and haemoglobin level, patient’s age (OR: 1.08, 95% CI: 1.03–1.13), GFR (OR: 0.94, 95% CI: 0.90–0.98), CKD stage 4 (OR: 24.66, 95% CI: 3.63–167.63) or CKD stage 5 (OR: 7.73, 95% CI: 1.63–27.27), spot urine PCR (OR: 1.83, 95% CI: 1.07–3.12), severe proteinuria (OR: 6.27, 95% CI: 1.66–23.60), no hypertension (OR: 0.09, 95% CI: 0.02–0.46), systolic blood pressure (OR: 1.04, 95% CI: 1.01–1.07), ULT (OR: 4.99, 95% CI: 1.54–16.16), loop diuretics (OR: 3.39, 95% CI: 1.01–11.42), obesity (OR: 6.12, 95% CI: 1.15–32.55) and no anaemia (OR: 0.04, 95% CI: 0.00–0.46) were significantly associated with hyperuricemia. Table 2.

## Discussion

Our study showed that more than half of CKD patients had hyperuricemia. Among a wide range of demographic, clinical and laboratory parameters evaluated, patient’s age, eGFR, spot urine PCR, hypertension, ULT, loop diuretics and anaemia were significantly associated with serum uric acid and hyperuricemia in CKD.

Hyperuricemia is common in CKD and mostly results from decreased uric acid excretion [10]. The kidneys play a major role in excreting uric acid, an end-product of purine metabolism [28]. We found a higher prevalence of hyperuricemia in CKD in our setting, compared to other SSA settings such as Nigeria [26] and Chad [27]. These divergent results can be largely attributed to study methodology. In the Nigerian study, the authors evaluated 120 pre-dialysis CKD patients and reported a prevalence of 47.5%. However, they excluded patients with gouty arthritis and patients on ULT. In the Chadian study, after evaluating 712 CKD patients, an even lower prevalence of 15.2% was reported. However, in this Chadian study, no information was provided on whether patients were on maintenance dialysis or not. Our higher prevalence could also be due to the high proportion of advanced CKD (73.2% CKD stages 4 and 5) amongst our study participants. Our prevalence was similar to the 70% reported in a paediatric population of CKD patients in USA [42]. The high proportion of advanced CKD amongst our study participants is due to the fact that our study was conducted in referral nephrology units which receive advanced cases of CKD from peripheral nephrology units.

We found significant association between eGFR, spot urine PCR and serum uric acid. Patients with CKD stages 4 or 5 and had 24.66 and 7.73 times higher significant odds respectively, of having hyperuricemia compared to those with CKD stage 3b. Also, patients with severe proteinuria had 6.27 significant higher odds of having hyperuricemia compared to those with no or mild proteinuria. These findings were similar to the results of Krishnan [43]. We also found a significant association between loop diuretics and serum uric acid in our sample of non-dialysed CKD. It is known that, loop diuretics predisposes individuals to hyperuricemia [44]. Diuretics cause hyperuricemia by increasing urate absorption and decreasing urate secretion in the kidneys [44]. Loop diuretics used in the management of hypertension and fluid retention in CKD should therefore be used judiciously.

We found a significant independent association between hypertension and hyperuricemia. Noone et al.*.* also reported a significant association between hypertension and hyperuricemia in paediatric patients with CKD [42]. A growing body of evidence suggest uric acid levels play a role in the development of hypertension [45, 46]. Uric acid can cause hypertension by mediating pro inflammatory pathways in vascular smooth muscles, inhibition of endothelial nitric oxide and activation of the renin-angiotensin system [45, 46]. On the other hand, hypertension increases renal vascular resistance, reducing renal flow, those increasing urate reabsorption [30]. Also, use of antihypertensive drugs like diuretics can increase serum uric acid levels [44]. It is however unlikely that in our study, the observed association between hypertension and hyperuricemia is due to use of diuretics as this was adjusted for in our analysis. Sedaghat et al.*.* suggested that hypertension mediates the decline in renal function caused by hyperuricemia [47]. Further research is needed to clarify the relationship between hyperuricemia and hypertension in CKD patients.

Subjects who were obese had 6.12 times significant higher odds of having hyperuricemia. This was in accordance with findings by Noone et al, in their paediatric CKD population [42]. Hyperuricemia seen in obesity has been attributed to insulin resistance and higher leptin production [30]. Insulin resistance in obese individuals causes larger amounts of insulin to be secreted in order to maintain glucose metabolism. The kidney responds to this hyperinsulinemia by decreasing urate clearance [48]. Obesity may therefore be a cause of hyperuricemia in our subjects, but a causal relationship between these two variables was not evaluated in this study.

It is possible that the observed association between hyperuricemia and anaemia is just an incidental finding. Both conditions can be caused by the underlying CKD, and there may not be a direct relationship between the two. However, in our analysis, after adjusting for eGFR and spot urine PCR, we still found a significant association between both variables. CKD patients with no anaemia displayed 0.04 times lower odds of having hyperuricemia after relevant confounder adjustment. To the best of our knowledge, there has been no study to depict the relationship between hyperuricemia and anaemia in CKD. In the Artherosclerosis Risk in the Communities (ARIC) study, McAdams-DeMarco et al. [49], after following up 10,791 individuals for a period of 9 years, reported that anaemia was associated with an approximately two-fold increased risk of ‘self-reported’ gout independent of renal function. The authors had no clear biological explanation for the link between anaemia and hyperuricemia, but hypothesised that this link may be mediated by oxidative stress. Increased oxidative stress seen in anaemia could cause hyperuricemia by increasing xanthine oxidase activity and increased cell death/turnover [50]. More research is needed to explicate this relationship.

Our study had strengths and limitations. It was well designed to capture a wide range of demographic, clinical and biological variables that could influence hyperuricemia in CKD. Furthermore, important variables such as medications and diet that could influence our findings were taken into consideration and accounted for. However the small sample size may have limited statistical power of some analyses. Also, odds ratios on logistic regression overestimate relative risks when the prevalence is high. In addition, the cross-sectional design did not enable us carryout cause and effect analysis for the various associations seen. This was particularly true for the associations seen between hypertension, obesity and anaemia with hyperuricemia.

## Conclusions

In this sample of non-dialysed CKD followed-up in referral centres in Cameroon, about 7 out of 10 had hyperuricemia. Hyperuricemia was independently associated with patient’s age, eGFR, spot urine PCR, hypertension, ULT, loop diuretics, obesity and anaemia. More studies are required to establish causal relationships between these associations.

## Abbreviations

BMI: Body mass index
CKD: Chronic kidney disease
DGH: Douala General Hospital
eGFR: Estimated glomerular filtration rate
ESRD: End-stage renal disease
HDL-C: Serum high-density lipoprotein cholesterol
IIQR: Interquartile ranges
LDL-C: Serum low-density lipoprotein cholesterol
PCR: Spot urine protein-creatinine ratio
RRT: Renal replacement therapy
SD: Standard deviation
SSA: Sub-Saharan Africa
ULT: Urate lowering therapy
YCH: Yaoundé Central Hospital
YUTH: Yaoundé University Teaching Hospital
